# One Health and the Politics of Antimicrobial Resistance

**DOI:** 10.3201/eid2304.161871

**Published:** 2017-04

**Authors:** Jean B. Patel

**Affiliations:** Centers for Disease Control and Prevention, Atlanta, Georgia, USA

**Keywords:** One Health, antimicrobial, antibacterial, antibiotic, antimicrobial resistance, drug resistance, drugs, Campylobacter, vancomycin-resistant enterococci, VRE, avoparcin, antimicrobial drugs

The use of antimicrobial drugs in food-producing animals can result in antimicrobial drug–resistant infections in humans. A good example is the use of fluoroquinolone in poultry and the emergence of fluoroquinolone-resistant *Campylobacter* infections among humans. However, topics of intense debate include the widespread use of antimicrobial drugs in animals, the extent to which antimicrobial drug use in animals affects human health, what drugs used in animals could be cause for concern to humans, and how to prevent overall risks to human health. 

The issue is complex from both policy and science perspectives. Antimicrobial drugs are used to raise all types of food-producing animals, but detailed drug use data are still lacking, making efforts to change drug use practices particularly challenging. In addition, because mobile genetic elements can confer new resistance on bacteria that already have complex ecology, there is often a lack of direct evidence to link the use of a particular antimicrobial drug to a specific resistant organism in humans. 

Policies that drive antimicrobial drug use, or lack of use, in animals can have profound effects on the health of the world’s population, the health of the agricultural industry, and the world’s food supply. However, even those well-versed in the topic of antimicrobial drug resistance find the issues of antimicrobial drug use in food-producing animals to be confusing and loaded with contrary political opinions on the significance of the public health threat and how best to address it.

Laura H. Kahn’s book One Health and the Politics of Antimicrobial Resistance (Figure) should be considered an essential primer for anyone who chooses to grapple with this challenging but crucial public health issue. The book is a concise summary of events and milestones that have been driving forces in the use of antimicrobial drugs for food production efforts and objectively outlines the effect these efforts have had on the problem of drug resistance. For example, few are aware of the effect World War II had on supply chains of traditional animal feed supplements such as cod liver oil and fishmeal. In the United States, this interruption resulted in the transition to using antimicrobial drugs to boost the growth of food-producing animals. The author not only provides the US perspective but also describes the history of antimicrobial drug use in Europe, the steps these countries have taken to curb the tide of drug resistance, and where progress has been made. An example is the decrease in vancomycin-resistant enterococci carriage among healthy people in the Netherlands and Germany after avoparcin was banned. 

**Figure:**
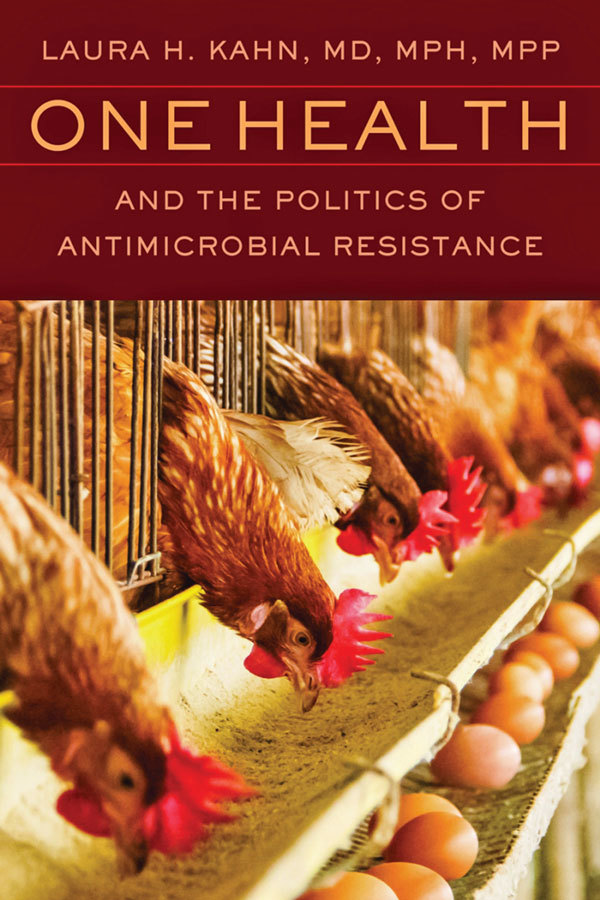
One Health and the Politics of Antimicrobial Resistance, Laura H. Kahn. John Hopkins University Press, Baltimore, MD, USA

The author demonstrates her command of both the politics and the science of establishing medication guidelines throughout the book and approaches the subject with professional objectivity. In the concluding chapter, she provides concrete recommendations for policy, surveillance systems, and scientific research to understand and prevent antimicrobial resistance from a One Health perspective.

